# Report of Surgical Cases

**Published:** 1862-10

**Authors:** Geo. K. Amerman

**Affiliations:** Attending Surgeon to the Chicago City Hospital


### CHICAGO MEDICAL JOURNAL.

VOL. VI.] OCTOBER, 1862. [No. 10.

### ORIGINAL COMMUNICATIONS.

### REPORT OF SURGICAL CASES

TREATED BY DR. GEO. K. AMERMAN,

*Attending Surgeon to the Chicago City Hospital.*

CASE I.—*Necrosis of the Entire Shaft of Femur—Amputation at Hip Joint—Death on the Seventh Day.*

A. E——, aged 16, born in Germany of healthy parents, was seized about the middle of January with a severe pain in the left knee. Previous to this he had enjoyed good health. This pain made its appearance suddenly, without any cause, and was exceedingly severe. It was soon followed by swelling and active febrile reaction. An intelligent surgeon was consulted, who at once recognized the nature of the difficulty and employed every means to arrest its progress. At a later period, an abscess formed just above the knee, which was opened and discharged a large quantity of pus. Notwithstanding every effort made to arrest the disease it soon became evident that the Femur was seriously affected. The abscess continued to discharge large quantities of unhealthy pus; the pain only partially subsided, and the febrile action continued, though less active. Three months after the first attack, I was called in consultation with his attending surgeon, Dr. Messler.

At this time the limb presented a very serious appearance. The leg and foot were œdematous and rotated inward so that the foot rested entirely on its inner side, the toes pointing toward the right instep. Commencing at the knee and extending upwards to within three inches of the Hip Joint, the limb was immensely swollen, and marked over the surface with large tortuous veins. One large senius existed at the inner and lower end of the thigh. The swelling terminated abruptly above and below, and presented an appearance exactly similar to malignant disease. Hectic fever every afternoon, diarrhœa, night sweats, tympanitis, anorrexia and emaciation were all present.

The only treatment that seemed to offer any prospect of relief was the removal of the limb, and although the chances of such an operation were extremely small, the patient readily assented and desired it.

There were present Drs. Hoffman, Messler, Smith, Heydock and Ross, who, together with myself, thought best to perform the Antero-Posterior flap operation high up, above the tumor, and if the bone was found healthy not to interfere with the joint. The operation was performed in the usual way, the patient being under the influence of chloroform. On sawing through the femur it was found enlarged, softened, and filled with a red grumous material, easily broken down under the probe and partially denuded. Under these circumstances it was thought best to remove the remainder of the shaft and head. This was accomplished by making one straight incision from the angle of union of the flaps on the outer side along the middle length of the bone and extending as high as the upper border of the acetabulum, severing the attachments of the muscles and ligaments and turning the head of the bone out of the socket. Dr. Hoffman compressed the femoral and controlled the hemorrhage so perfectly that not over six ounces of blood was lost during the operation. Dr. Messler took charge of the case after the operation, and from his report I learn that everything progressed favorably for five days, but on the sixth day he began to sink and died the next day from exhaustion. It is proper to remark that the surroundings of this patient were of the most unfavorable character. Beef tea, brandy, aud constant care were not to be had, else, possibly, the result might have been different. On examining the limb, after removal, the disease was found to extend throughout the entire length of the femur. The lower end was fractured transversely just above the condyles, and what remained of the lower fragment was loosely adherent to the head of the tibia and overlying the lower end of the upper fragment. The remainder of the shaft was denuded, rough, and perforated in several places. New bone material had been poured out in the shape of long slender spicula supporting each other and forming a kind of bridge, extending from the shaft of the old bone to the head of the tibia, thus binding the two together.

The points of interest in connection with the above case are few and simple: First, we notice the absence of any cause. Constitutionally, the patient seemed in no way predisposed to necrosis. He had no appearance of a scrofulous or syphilitic taint, and had always enjoyed excellent health. No local cause, so far as we could ascertain, produced it. In the second place, it will be observed that the disease pursued an unusually rapid course. In three months after its first appearance the entire shaft of the femur, the longest bone in the body, was necrosed. And again, the *extent* of the disease. The shaft, both extremities, and the whole thickness of the bone were affected. When necrosis affects the long bones it is generally confined to one of the extremities, or to a portion of the shaft, but in this case we had the bone *entire* affected, without any apparent cause, and terminating in its complete destruction in three months.

---

CASE II.—*Amputation of Thigh—Secondary Hemorrhage on the Ninth Day Controlled by Pressure and Position—Recovery.*

J. H——, aged twenty-five, in good health, with no predisposition to disease, was injured by a railroad car on Tuesday, June 20th. He received the injury in the morning and was immediately taken to the City Hospital. I saw him in the early part of the afternoon, and found, on examination, a compound comminuted Fracture of the right Tibia and Fibula, with some contusion of the soft parts, and probable laceration of the Ant. Tibial Artery. The right leg and foot were cold, livid, and pulseless. Reaction was well established, and everything seemed to require and render favorable an immediate amputation. The patient was placed under the influence of chloroform and the limb removed in lower third of thigh by the ordinary flap method. Simple dressings, with constant irrigation of cold water, was used for four days. On the fifth day, the stump was redressed and presented every appearance of a favorable union. On the ninth day, whilst the patient and his attendant were asleep, secondary hemorrhage occurred, and he lost nearly a quart of blood before it was discovered. As soon as discovered, compression was made over the femoral, which entirely controlled it. I saw him about one hour afterwards. He was blanched and pale; pulse 120, and weak. Ordered brandy and opium, and as soon as he got over the excitement and fright occasioned by so unexpected and rapid loss of blood, proceeded to remove the dressings. The dressings and blood clots were all carefully removed, the stump washed in cold water, and elevated. There was no recurrence of the hemorrhage, though the pressure from the femoral was removed. From this time forward the case progressed favorably and the patient was discharged cured August 29, ten weeks after the amputation.

There is nothing in the treatment of the above case worthy of notice; but the favorable termination of a case of *primary* amputation *of the Thigh* for *railroad* injury renders it of considerable interest and importance. It is said, that not a single case of primary amputation of the thigh, after one of the late battles, recovered, and this coincides with my own experience of the operation performed for railroad injuries. The causes of this large fatality have often been discussed, and much information elicited, but none satisfactorily explained. Another point of some interest in connection with the above case, is the occurrence of secondary hemorrhage. This is among the most frequent secondary accidents after amputations of the thigh, and perhaps one of the most annoying, if not serious. I have had two cases during the last year. Both were in amputations of the thigh and both were permanently arrested by pressure and position. I should expect in every case to succeed by these means alone. The pressure must be made by the fingers of an assistant, who is to keep constant watch over the patient day and night, until all danger from this source has passed away.

